# Divergent Anabolic Signalling responses of Murine Soleus and Tibialis Anterior Muscles to Chronic 2G Hypergravity

**DOI:** 10.1038/s41598-017-03758-x

**Published:** 2017-06-14

**Authors:** Timur Mirzoev, Sergey Tyganov, Irina Petrova, Vasily Gnyubkin, Norbert Laroche, Laurence Vico, Boris Shenkman

**Affiliations:** 1 Myology Laboratory, Institute of Biomedical Problems RAS, Moscow, Russia; 2 INSERM U1059, Integrative Biology of Bone Tissue Laboratory, Lyon University, St.- Etienne, France

## Abstract

The purpose of the study was to assess the rate of protein synthesis (PS) and elucidate signalling pathways regulating PS in mouse soleus (Sol) and tibialis anterior (TA) muscles following chronic hypergravity (30-day centrifugation at 2G). The content of the key signalling proteins of the various anabolic signalling pathways was determined by Western-blotting. The rate of PS was assessed using *in-vivo* SUnSET technique. An exposure to 2G centrifugation did not induce any significant changes in the rate of PS as well as phosphorylation status of the key anabolic markers (AKT, p70s6k, 4E-BP1, GSK-3beta, eEF2) in Sol. On the contrary, a significant 55% increase in PS (p < 0.05) was found in TA. The cause of such a rise in PS could be associated with an increase in AKT (+72%, p < 0.05), GSK-3beta (+60%, p < 0.05) and p70s6k (+40%, p < 0.05) phosphorylation, as well as a decrease in eEF2 phosphorylation (−46%, p < 0.05) as compared to control values. Thus, the results of our study indicate that 30-day 2G centrifugation induces a distinct anabolic response in mouse Sol and TA muscles. The activation of the PS rate in TA could be linked to an up-regulation of both mTORC1-dependent and mTORC1-independent signalling pathways.

## Introduction

Skeletal muscle is a remarkably plastic tissue that is capable of responding to different physical stimuli. One of the factors that can induce significant changes in a mammalian skeletal muscle is a level of gravity. It has long been established that an exposure to real or simulated microgravity results in a pronounced atrophy and reduced functional capacity of slow postural muscles such as the soleus (Sol)^1–5^. It is believed that unloading-induced reduction in skeletal muscle mass and functional capacity could be prevented by application of artificial gravity via centrifugation. It has been shown that artificial gravity (1G) created by centrifugation during space flight can partly prevent a decrease in the cross-sectional area (CSA) of rat soleus muscle^6^. D’Aunno *et al*.^7^ demonstrated that intermittent centrifugation can partially maintain the mass of rat Sol during unloading. Caiozzo *et al*.^8^ showed that daily 2.5G centrifugation led to an attenuation of torque loss of plantarflexor and knee extensor muscles as well as a full prevention of Sol muscle atrophy during 21-day bed rest. However, despite the considerable knowledge gained in space physiology, the effectiveness of onboard countermeasures is still not fully clarified^9^. Moreover, molecular mechanisms involved in the regulation of protein synthesis (PS) and muscle mass under conditions of hypergravity remain unexplored. To date, it is well known that skeletal muscle mass in response to increases/decreases in loading is critically regulated by the activation of the serine/threonine kinase, the mammalian Target Of Rapamycin Complex 1 (mTORC1), resulting in increases in mRNA translation initiation and ribosome biogenesis^10–13^. The main effectors of mTORC1 are ribosomal protein kinase p70 (p70s6k) and translation initiation factor 4E binding protein (4E-BP1)^14^. In addition, translation initiation can be regulated via glycogen synthase kinase 3β (GSK-3β), which is a key element of AKT/GSK-3β/eIF2B signalling pathway. Protein kinase B (AKT) can phosphorylate and inhibit GSK-3β^15^, thereby activating translation initiation factor 2B (eIF2B)^16^. mTORC1-signalling can be regulated via Adenosine Monophosphate Activated Protein Kinase (AMPK), since AMPK activation can lead to a decrease in mTOR activity^17, 18^. The key marker of AMPK activity is its downstream target, Acetyl-CoA Carboxylase (ACC)^19^. Another signalling molecule that is essential in mRNA translation and can be modified following altered gravitational environment is eukaryotic elongation factor 2 (eEF2). eEF2 is able to inhibit translation elongation being phosphorylated on the Thr56 residue by eEF2 kinase^20^. To date, to our knowledge, there is a lack of data concerning the activity of anabolic signalling pathways as well as the rate of PS in rodent skeletal muscle following an exposure to chronic hypergravity. Therefore, the purpose of the present study was to measure the rate of PS and the activity of the key markers of various anabolic signalling pathways in mouse Sol and tibialis anterior (TA) following 30-day centrifugation at 2G. We hypothesized that an exposure to chronic hypergravity would activate anabolic signalling pathways with subsequent rise in PS in mouse skeletal muscles. At the same time, it is well-known that neuromuscular activities of dorsal extensors and flexors can differ under hypergravity conditions^21–23^. Therefore, we aimed to compare anabolic signalling responses in two hindlimb muscles, i.e. dorsal extensor (Sol) and dorsal flexor (TA) after chronic 2G hypergravity.

## Materials and Methods

### Animals and centrifugation

The experiments were carried out on male C57BL6J mice (body weight ~25.5 g) obtained from Charles River, France. The animals were randomly divided into a control (Con, n = 8) group and a group submitted to a 30-day 2G centrifugation (2G group, n = 8). The 2G group was placed on a 2.8-m-diameter centrifuge. To obtain 2G environment, 29.6 rpm was applied. The 2G animal cages were housed inside centrifuge gondolas, which provided ventilation, a 24-h light-dark cycle (light-dark 12:12 h) and an ambient temperature of 22 ± 1 °C. Food and water were available *ad libitum*. The Con mice were kept in conditions similar to those of the centrifuge, i.e., the same room, same dark-light cycle (12:12 h), and same temperature inside a standard home cage contained in a gondola. At the end of the 2G exposure, the mice were removed from the centrifuge and euthanized by cervical dislocation, and soleus and tibialis anterior muscles were excised, weighed, then snap-frozen in liquid nitrogen, and stored until analyzed.

The experiments and the maintenance conditions of the animals were approved by both the Agricultural and Forest Ministry of France (authorization 04827) and local Animal Care Committee of the Lyon University, France. All experiments were performed in strict accordance with the guidelines and recommendations in the Guide for the Care and Use of Laboratory Animals of the National Institutes of Health. All efforts were made to minimize animal suffering and discomfort and to reduce the number of animals used.

### SUnSET technique for measuring the rate of protein synthesis

SUnSET (surface sensing of translation) is a nonradioactive technique that allows to measure protein synthesis *in vivo* in skeletal muscle. This technique involves the use of the antibiotic puromycin (a structural analog of tyrosyl-tRNA), and anti-puromycin antibodies to detect the amount of puromycin incorporation into nascent peptide chains^13^. It was shown that when puromycin is used at low concentrations (40 nmol/g), the accumulation of puromycin-conjugated peptides accurately reflects the rate of protein synthesis^24^. SUnSET technique uses standard Western blotting and immunohistochemical technologies to visualize and quantify *in vivo* rates of protein synthesis^13^. For *in vivo* measurements of protein synthesis, Con and 2G mice were given an intraperitoneal injection of 40 nmol/g puromycin hydrochloride (Enzo Life Sciences, NY, USA) dissolved in PBS. At exactly 30 min after injection, muscle tissue was extracted and frozen in liquid nitrogen for WB analysis.

### Western blot analysis

**Figure 1:**
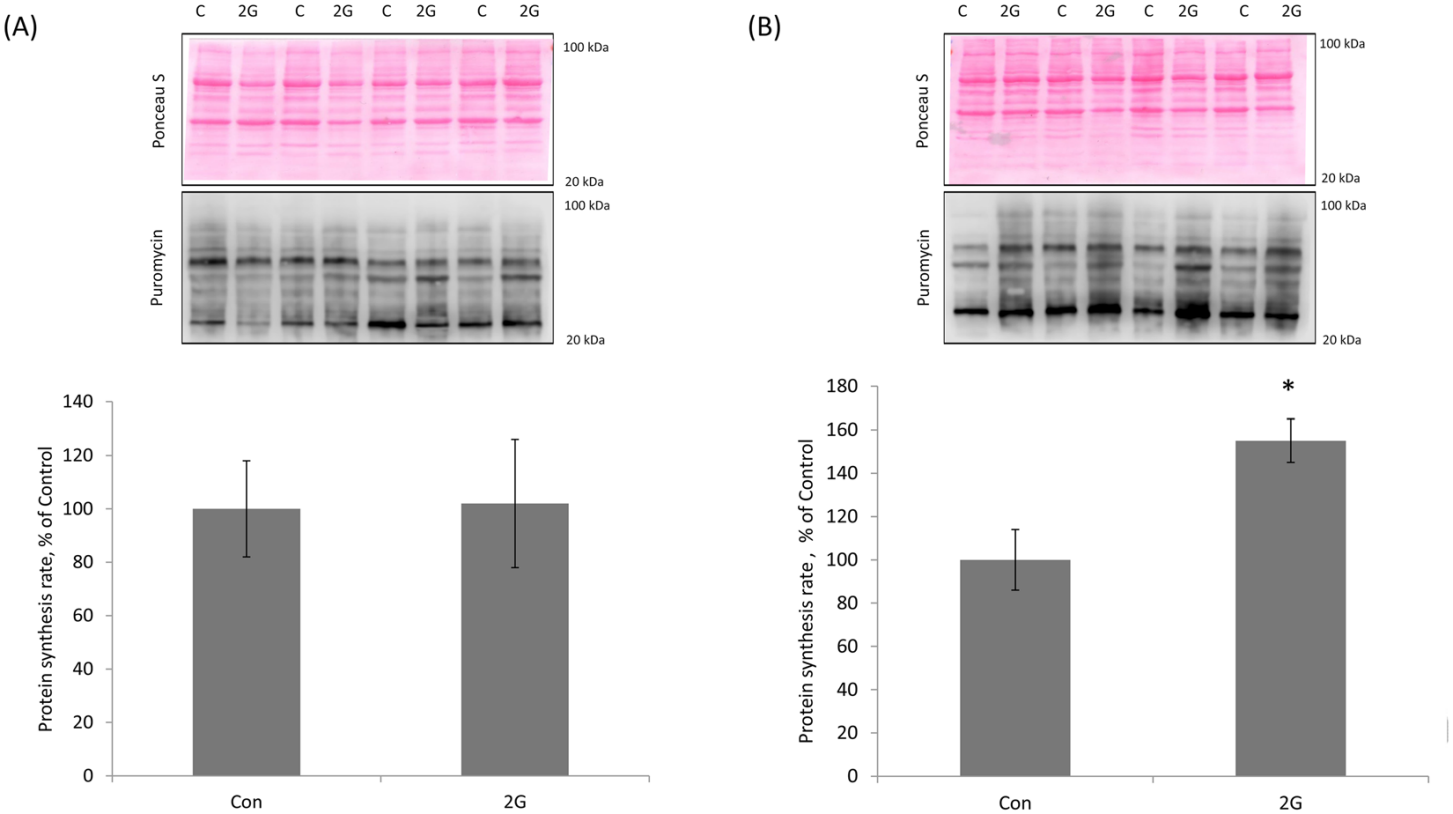
The rate of protein synthesis in murine Sol (**A**) and TA (**B**) muscles following 30-day 2G centrifugation. Quantification of the puromycin-labeled peptides is expressed as a percentage of the values obtained in the control group. Representative WB images for puromycin and Ponceau S staining to verify equal loading of proteins are shown above the graphs. Con – control mice, 2G – mice exposed to 30-day 2G centrifugation. *Significant difference from control (p < 0.05). All values are means ± SE, n = 8/group.

**Figure 2:**
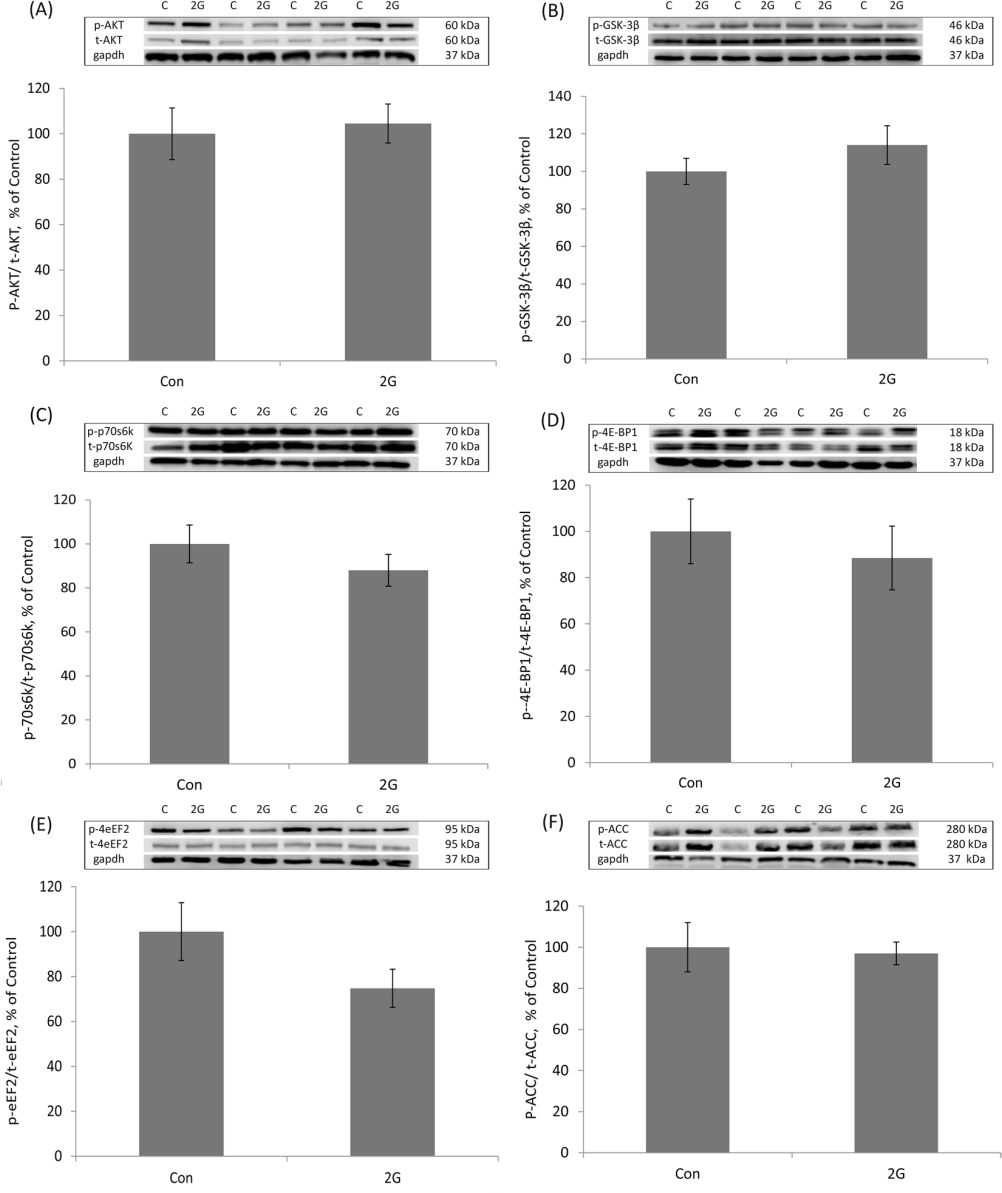
Phosphorylation status of the key anabolic signalling proteins in murine soleus (Sol) muscle following 30-day 2G centrifugation. (**A**) Quantification of phospho-AKT/total AKT ratio, expressed relative (%) to control. Representative blots are shown above the graph. (**B**) Quantification of phospho-GSK-3β/total GSK-3β ratio, expressed relative (%) to control. Representative blots are shown above the graph. (**C**) Quantification of phospho-p70s6k/total p70s6k ratio, expressed relative (%) to control. Representative blots are shown above the graph. (**D**) Quantification of phospho-4E-BP1/total 4E-BP1 ratio, expressed relative (%) to control. Representative blots are shown above the graph. (**E**) Quantification of phospho-eEF2/total eEF2 ratio, expressed relative (%) to control. Representative blots are shown above the graph. (**F**) Quantification of phospho-ACC/total ACC, expressed relative (%) to control. Representative blots are shown above the graph. Con – control mice, 2G – mice exposed to 30-day 2G centrifugation. *Significant difference from control (p < 0.05). All values are means ± SE, n = 8/group.

**Figure 3:**
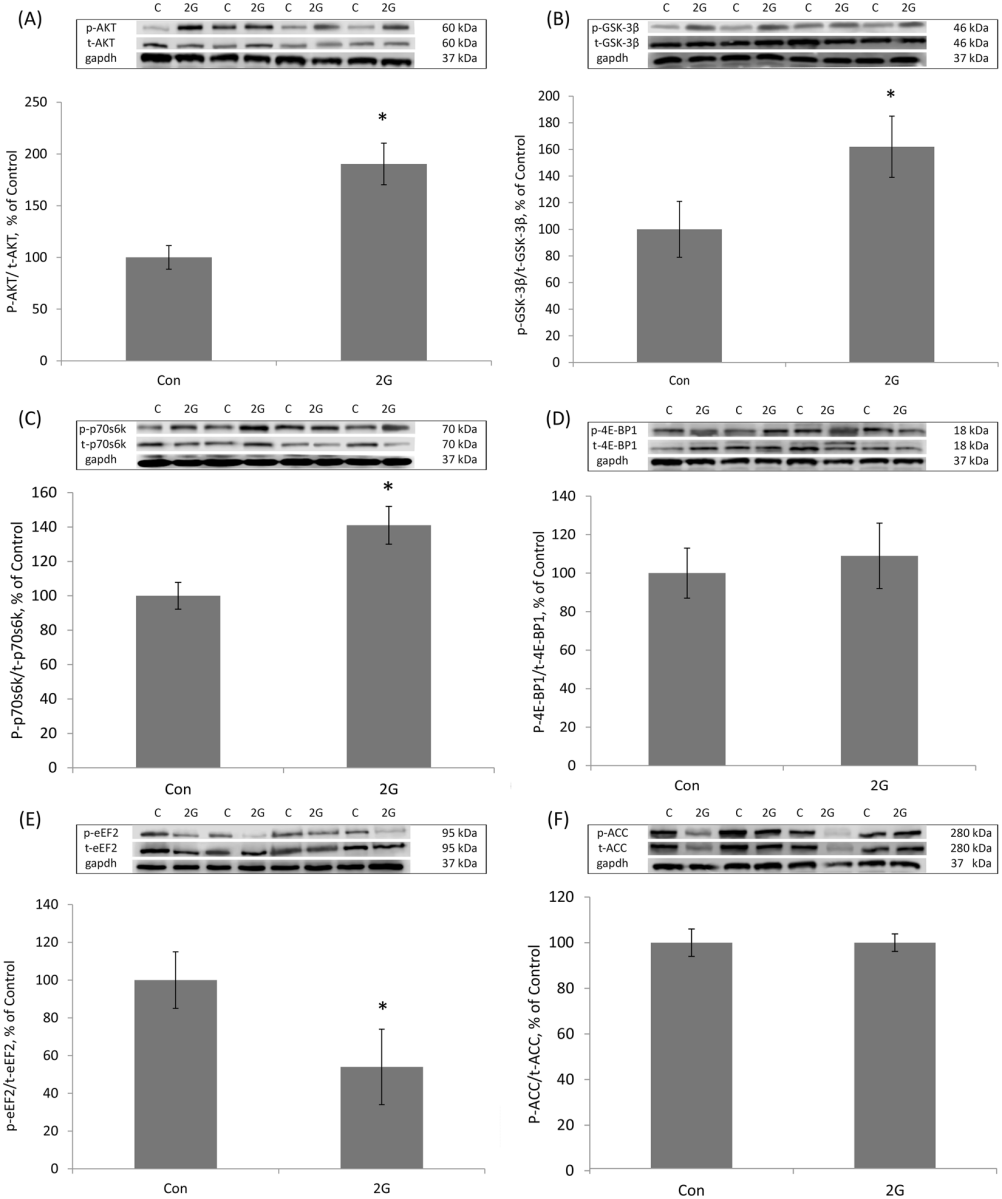
Phosphorylation status of the key anabolic signalling proteins in mouse tibialis anterior (TA) muscle following 30-day 2G centrifugation. (**A**) Quantification of phospho-AKT/total AKT ratio, expressed relative (%) to control. Representative blots are shown above the graph. (**B**) Quantification of phospho-GSK-3β/total GSK-3β ratio, expressed relative (%) to control. Representative blots are shown above the graph. (**C**) Quantification of phospho-p70s6k/total p70s6k ratio, expressed relative (%) to control. Representative blots are shown above the graph. (**D**) Quantification of phospho-4E-BP1/total 4E-BP1 ratio, expressed relative (%) to control. Representative blots are shown above the graph. (**E**) Quantification of phospho-eEF2/total eEF2 ratio, expressed relative (%) to control. Representative blots are shown above the graph. (**F**) Quantification of phospho-ACC/total ACC, expressed relative (%) to control. Representative blots are shown above the graph. Con – control mice, 2G – mice exposed to 30-day 2G centrifugation. *Significant difference from control (p < 0.05). All values are means ± SE, n = 8/group.

The skeletal muscle tissue was homogenized in the ice-cold lysis buffer: 50 mM Tris (pH 7.4), 150 mM NaCl, 1% Nonidet P-40, 0.5% sodium deoxycholate, 0.1% SDS, 0.004% sodium azide, and 5 mM EDTA, supplemented with 1 mM DTT, 1 mM PMSF, 10 μg/ml leupeptin, 5 μl/ml pepstatin and 1% aprotinin (Sigma-Aldrich, MO, USA), mammalian protease inhibitor cocktail (Amresco, Solon, OH, USA), and phosphatase inhibitor cocktail B (Santa Cruz Biotechnology, CA, USA). The total protein concentration of the lysates was determined by incubation for 20 min at 4 °C and centrifugation for 10 min at 12,000 g. The protein content of the supernatants was quantified using an assay based on a modification of the Lowry protocol (RC DC Protein Assay; Bio-Rad Laboratories, Hercules, CA, USA). Bovine serum albumin was used as a standard. The samples were diluted in Laemmli buffer. The total protein (20–50 μg) was subjected to SDS-PAGE, and the proteins were then transferred to nitrocellulose membrane (Bio-Rad Laboratories, CA, USA). Then, to verify equal loading of protein in all lanes, the nitrocellulose membrane was dyed by Ponceau S. The membranes were blocked for 1 h at room temperature with the blocking buffer (4% nonfat milk powder; TBS, pH 7.4; and 0.1% Tween 20) and incubated overnight at 4 °C with primary antibodies (diluted in TBS-T) against p-4E-BP1 Thr37/46 (1:1000, Cell Signaling Technology, Beverly, MA, USA, #2855) and t-4E-BP1 1:1000; Cell Signaling Technology, Beverly, MA, USA, #9452); p-p70s6k Thr389 (1:2000, Santa Cruz Biotechnology, USA, sc-11759) and t-p70s6k (1:1000, Cell Signaling Technology, USA, #9202); p-AKT Ser 473 (1:1000, Cell Signaling Technology, USA, #4058) and t-AKT (1:1000, Cell Signaling Technology, USA, #9772); p-GSK-3β Ser9 (1:1000, Cell Signaling Technology, USA, #9322) and t-GSK-3β (1:1000, Cell Signaling Technology, USA, #12456); p-eEF2 Thr56 **(**1:1000, Cell Signaling Technology, USA, #2331) and t-eEF2 **(**1:1000, Cell Signaling Technology, USA, #2332); p-ACC Ser79 (1:1000, Cell Signaling Technology, USA, #3661), t-ACC (1:2000, Cell Signaling Technology, USA, #3662), puromycin (1:3000; Kerafast Inc., Boston, MA, USA, catalog # EQ0001) and glyceraldehyde-3-phosphate dehydrogenase (GAPDH, 1:10000; Applied Biological Materials Inc., Canada, #G041). Three 10-min washes with TBS-T were then performed. After that, the membranes were incubated for 1 h at room temperature with horseradish peroxidase-conjugated secondary antibodies to rabbit or mouse immunoglobulins (diluted 1:200,000; Bio-Rad Laboratories, CA, USA). The membranes were then washed again in TBS-T 3 times for 10 min and incubated in Immun-Star HRP Chemiluminescent system (Bio-Rad Laboratories, Hercules, CA, USA). The protein bands were quantified using C-DiGit Blot Scanner (LI-COR Biotechnology, USA) and Image Studio Digits software. Following image capture of phosphorylated proteins, membranes were stripped of the phosphospecific antibodies, using Restore^TM^ Western Blot Stripping Buffer (Thermo Scientific, USA), for 30 min at 37 °C after which the membranes were re-probed with primary antibodies for each respective total protein. For protein synthesis detection, the measurements of the chemiluminescent signals were performed by determining the density of each whole lane with the entire molecular weight range of puromycin-labeled peptides. Phospho and total blots were done on the same gel. Each gel contained samples from both C and 2G group. Protein lysate samples from skeletal muscles of C and 2G mice were run consecutively on the same gels to standardize exposure times between groups. Phosphorylation status as a proxy of activation of the signaling proteins was expressed relative to the total amount of each protein. Protein samples were run at least in duplicate on the same gel. The representative blots are of the same samples (phospho and total). Total protein staining (Ponceau S) and GAPDH protein expression were used as loading controls. Representative images of Ponceau staining (Fig. 1) and GAPDH blot (Figs 2 and 3) show that protein loading did not change between groups.

### Statistical analysis

All data are expressed as means ± SEM. For the two muscles, after a one-way ANOVA, the intergroup comparisons were performed using Student’s t-test with a significance level of p < 0.05.

## Results

### Body mass and muscle weights

Table 1 shows that a period of 30-day 2G centrifugation did not induce a significant change in body weight compared to 1G control. An absolute and body mass-adjusted soleus weights from 2G group did not differ from Con group. By contrast, a period of chronic hypergravity resulted in a significant increase (p < 0.05) in both absolute and body mass-adjusted weights of tibialis anterior muscle (Table 1).

**Table 1:** Body mass and absolute and body mass-adjusted wet weights of the soleus (Sol) and tibialis anterior (TA) muscles.

Group	Control group (1G)	2G group
Weight		
Initial body mass, g	25.4 ± 0.32	26 ± 0.27
Final body mass, g	26.7 ± 0.9	30.2 ± 0.61
Sol wet weight, mg	9.45 ± 0.38	10.3 ± 0.61
Sol wet weight/body weight, mg/g	0.34 ± 0.012	0.35 ± 0.011
TA wet weight, mg	37 ± 4.8	50 ± 3*
TA wet weight/body weight, mg/g	1.15 ± 0.15	1.73 ± 0.11*

Values are means ± SEM.; n = 8/group. *Significant difference *vs*. Control, p < 0.05.

### The rate of protein synthesis

In Sol muscle, we did not observe any significant changes in the rate of PS between 1G and 2G groups (Fig. 1A). However, SUnSET measurements revealed a significant 55% (p < 0.05) increase in the PS rate in TA following an exposure to 2G centrifugation (Fig. 1B).

### The phosphorylation status of the key signalling molecules involved in the regulation of protein synthesis

In Sol muscle, an exposure to chronic hypergravity did not induce any significant alterations in the phosphorylation levels of the key anabolic markers, i.e. AKT (Fig. 2A), GSK-3β (Fig. 2B), p70s6k (Fig. 2C), 4E-BP1 (Fig. 2D), eEF2 (Fig. 2E). The phosphorylation status of the AMPK activity indicator, ACC, was not affected by 2G chronic centrifugation as well (Fig. 2F).

In TA muscle, Western blot analysis revealed a significant increase in phosphorylated/total ratios of AKT (+72%) (Fig. 3A), GSK-3β (+62%) (Fig. 3B), p70s6k (+41%) (Fig. 3C) and as well as decreased phosphorylation in eEF2 (−46%) (Fig. 3E) following 2G centrifugation. The phosphorylation levels of 4E-BP1 (Fig. 3D) and ACC (Fig. 3F) in TA remained unchanged.

## Discussion

To our knowledge, here we report, for the first time, the effect of 30-day 2G-centrufugation on the key markers of signalling pathways involved in the regulation of mRNA translation in murine Sol and TA muscles. We hypothesized that an exposure to chronic hypergravity would lead to alternations in phosphorylation of anabolic signalling molecules resulting in an increase in PS and subsequent hypertrophy of skeletal muscles.

We found that a period of 30-day 2G centrifugation did not affect both absolute and relative weight of the antigravity Sol muscle. Taking into account previously published papers, it appears that an exposure to hypergravity may have divergent effects on Sol muscle weight/cross-sectional area (CSA) of muscle fibres, depending on the level and duration of hypergravity as well as species, age and sex of the animals. Indeed, Oganov *et al*.^25^ did not observe any changes in rat Sol weight following 21-day 2G centrifugation^25^. Two-week 2G centrifugation did not induce any alternations in the mean fibre size of rat Sol^26^ or rhesus Sol muscle^27^. Vasques and colleagues reported that in 2G-exposed (for 2 weeks) male rats, absolute weights of predominantly slow muscles in the hindlimb were maintained^28^. It has been also shown that absolute rat Sol weight and CSA of Sol muscle fibres did not change following 33-day chronic hypergravity^29^. Fuller and co-authors also observed that absolute and body mass-adjusted rat Sol weights after 8-week 2G centrifugation did not differ from control^30^. In addition, it has been shown that in 100-day-old rats born and reared in 2G environment absolute Sol weight decreased while body mass-adjusted Sol weight remained unchanged^31^. All these data are in agreement with the result of the present study. On the other hand, Stevens *et al*.^32^ observed a 15% increase in 2G-exposed (19 days) rat Sol muscle mass, whereas the CSA of Sol fibres remained unaltered^32^. This increase in Sol weight is more likely associated with an increase in connective tissue^33^ or complexes of the extracellular matrix^34, 35^. There are reports showing that an exposure to greater levels of gravitational field (3G) for 4 or 14 weeks can result in a significant increase in mouse Sol weight as well as CSA of Sol muscle fibres^36, 37^.

One explanation for the lack of a hypertrophic response of Sol muscle could be the lack of changes in muscle PS. Therefore, we estimated both the rate of PS and the phosphorylation status of the key signalling molecules involved in the regulation of mRNA translation. Our results show that neither the rate of PS nor the phosphorylation level of p70s6k, 4E-BP1, GSK-3β, ACC and eEF2 were altered in mouse Sol following a period of 30-day hypergravity.

In contrast to the Sol, 30-day centrifugation induced a significant increase in the TA muscle (Sol antagonist). Indeed, both absolute and normalized to body mass TA weight was significantly higher after an exposure to 30-day centrifugation. Moreover, this increase in the TA muscle weight was accompanied by a significant rise in the rate of PS, as assessed by SUnSET technique. Our results suggest that an increase in PS in TA was linked to post-translational modifications of the key markers of both mTORC1-dependent and mTORC1- independent pathways. Specifically, we found a significant increase in AKT and p70s6k phosphorylation (an indicator of mTORC1 activity) as well as GSK-3β (a marker of mTORC1-independent GSK-3β/eIF2B pathway). GSK-3β can be phosphorylated and inactivated by AKT^15^ leading to facilitation of mRNA translation via eIF2B^16^. Consistent with the increase in AKT phosphorylation, GSK-3β phosphorylation was increased in TA following 2G-centrifugation. The phosphorylation of another mTORC1 substrate, 4E-BP1, remained unchanged. A divergent response of p70s6k and 4E-BP1 to hypergravity might be explained by differential regulation of these proteins. It has been shown that mTORC1 output to 4E-BP1 is rapamycin-insensitive whereas mTORC1 signalling to p70s6k is rapamycin-sensitive^38^. There is evidence that phosphoinositide-3-kinase (PI3K) and mTOR inputs to p70s6k can be separated. Deletion of an N-terminal p70s6k fragment confers rapamycin resistance to the p70s6k protein, yet this truncation mutant remains sensitive to treatment with PI3K inhibitors^39, 40^. Moreover, it has been showed that an increase in p70s6k phosphorylation can occur without a similar effect on 4E-BP1^41, 42^. A significant decrease in eEF2 phosphorylation in TA, which was observed in our study, could also contribute to the activation of PS via enhancing translation elongation. The phosphorylation level of the key indicator of AMPK activity, ACC, did not alter following 30-day 2G centrifugation in both Sol and TA muscles. This result is in agreement with Hilder *et al*.^43^ who showed that AMPK activity in rat Sol muscle was not affected by chronic 2G centrifugation. To the best of our knowledge, in our study, we report for the first time on signalling responses of murine TA muscle to a period of chronic 2G hypergravity.

There is evidence that exercise and chronic acceleration (hypergravity) produce similar adaptations in a skeletal muscle. For instance, both exercise and hypergravity can cause an increase in the slow myosin heavy chain isoform expression in rat Sol muscle, suggesting that loading is a primary stimulus for this shift^30^. We suppose that a divergent anabolic response of the Sol (ankle extensor) and TA (ankle flexor) could be associated with a distinct loading of the extensors and flexors under hypergravity conditions. Unfortunately, in the present study, an electromyographic (EMG) activity of the muscles during 2G centrifugation was not measured. However, it is well established that during hypergravity phases of parabolic flight, the EMG activity of the extensor muscles significantly increases, whereas the EMG level of flexors only slightly increases or remains unchanged^21–23^. It is known that during exercise/loading the rate of muscle PS is blunted or remains unchanged^44–46^. Thus, the lack of change in the Sol mass in the present study might be related to the unchanged muscle protein synthesis during enhanced loading caused by continuous 2G centrifugation. It can be also assumed that a chronic 30-day 2G centrifugation could be excessive for the postural muscle so that the Sol muscle would reach the limit of its adaptive capabilities resulting in a lack of anabolic response. This speculation is supported by the evidence that a chronic 2G centrifugation can lead to myofibrillar damage and increased number of nuclei with transcriptionally inactive highly condensed chromatin in rat soleus muscle^29, 33^. Another explanation is that TA, not being a postural muscle, in 1 G environment is not constantly loaded as compared to postural Sol muscle. In hypergravity conditions TA can become more susceptible to the altered gravity which would result in hypertrophy. It could be explained by the fact that in response to resistance loading, hypertrophy is more pronounced in fast-twitch (type II) than slow-twitch (type I) fibres^47–50^. In C57BL/6 J mice, TA is composed exclusively of fast-twitch fibres^51^ while Sol contains about 40% of slow-twitch fibres^51, 52^. The opposite reaction of TA and Sol is seen under real or simulated microgravity: ankle extensors (like Sol) atrophy to a greater extent than flexors (like TA)^1, 5, 53–55^.

In conclusion, the results of our study indicate that 30-day 2G centrifugation induces a distinct anabolic response in mouse Sol and TA muscles. The activation of the PS rate in TA could be linked to the up-regulation of both mTORC1-dependent and mTORC1-independent signalling pathways.
